# Surgical technique for the open repair of grade 3–4 tears of the gluteus medius tendon of the hip: Technical note and case series

**DOI:** 10.1016/j.ijscr.2025.111191

**Published:** 2025-03-24

**Authors:** Damien Van Quickenborne, Catherine Van Der Straeten, Arne Burssens, Emmanuel Audenaert

**Affiliations:** a ANCA Clinic Ghent, Xavier De Cocklaan 68/1, 9831 Deurle, Belgium; b Ghent University Hospital, Orthopaedic Department, Corneel Heymanslaan 10, 9000 Ghent, Belgium

**Keywords:** Hip, Augmentation, Tendon

## Abstract

**Introduction and importance:**

Greater Trochanteric Pain Syndrome (GTPS) affects 10–25 % of the population, predominantly middle-aged and elderly women. It significantly impacts quality of life, comparable to end-stage hip osteoarthritis. Gluteus Medius Tendon Tears (GTT) are a critical component of GTPS pathology.

**Case presentation:**

The study introduces an innovative open surgical technique for treating Grade III and IV Gluteus Medius (GMed) tendon tears, utilizing a polyethylene terephthalate (PET) synthetic ligament for augmentation. 42 patients are included in this case series.

**Discussion:**

Existing therapeutic surgical options for GTT are limited and challenging, with historically poor repair outcomes. The proposed technique using PET synthetic ligament represents a potential advancement in surgical management.

**Conclusion:**

The novel surgical approach offers a promising alternative for addressing complex Gluteus Medius tendon tears, potentially improving patient outcomes and functional restoration.

## Highlights

• Novel surgical technique for Gluteus Medius Tendon tears developed by single surgeon
• 42-patient case series at one center with 36 women and 4 men, average age 65.3 years
• Procedure involves synthetic ligament reinforcement.
• Pain scores significantly reduced from 7.9 to 2.7 at 6 months post-surgery.
• Multi-center research needed to validate technique's broader applicability

## 1 Introduction

Greater trochanteric pain syndrome (GTPS) is a relatively common cause of lateral hip pain, with an estimated prevalence ranging from 10 % to 25 % of the population, most often reported in middle-aged and elderly women. GTPS is a prevalent and severely debilitating disorder impacting work participation, physical activity and quality of life, and therefore it should be considered equally important as grade IV osteoarthritis [1].

Gluteus Medius Tendon Tears (GTT) are a significant contributor to GTPS. Furthermore, the prevalence of GTT among patients diagnosed with GTPS is likely underreported [2]. This might contribute to the suboptimal treatment strategies for a substantial subset of GTPS patients.

**Fig. 1:**
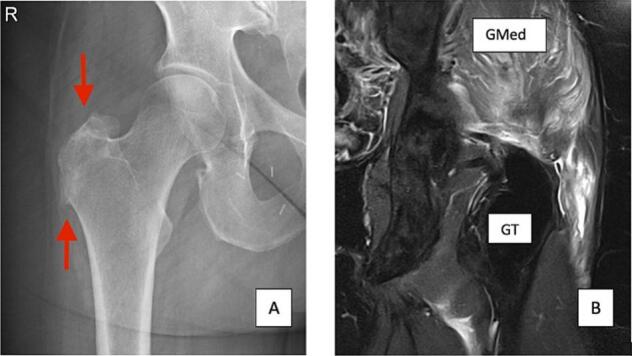
A) Standing AP Xray of the right hip with distal spur of the GT and irregular cortex (red arrows) B) T2 MRI left hip with fatty degeneration of the GMed and liquid. (For interpretation of the references to color in this figure legend, the reader is referred to the web version of this article.)

The awareness of the potential (sub)total GMed Tendon Tear (GTT) in patients with GTPS should be high. The diagnostic work-up should be standardized and involves: Standing AP Xray of the Pelvis, Ultrasound and Magnetic Resonance Imaging in accordance with the International Society of Hip Preservation (ISHA) guidelines [3]. Partial-thickness tendon tears are commonly described as an increased T2-weighted signal combined with thinning and/or focal discontinuity of the GMed tendon, whereas full-thickness tears imply complete discontinuity of the tendon fibers and/or retraction of the gluteal tendon [4]. There is always a substantial bursa around the retracted GMed Tendon on MRI. On standing Xray of the pelvis or the hip, a “reshaping” of the Greater Trochanter (GT) can be noticed. The lateral cortex of the GT becomes irregular in end-staged GTT, and a “spur” formation at the proximal and distal part of the lateral GT typically develops (Fig. 1).

Conservative treatment for GTT focuses on non-surgical interventions to reduce pain and improve function. This approach includes structured physical rehabilitation to strengthen gluteal muscles and enhance hip biomechanics. Patients are advised to modify activities that worsen symptoms. Pain management often involves oral analgesics and anti-inflammatory drugs. Peri-trochanteric injections, such as corticosteroids or platelet-rich plasma, may provide relief. Adjunct therapies like radial shockwave treatment can also be employed [3,5]. Conservative treatment for GTT, while often the initial approach, shows limited effectiveness for full-thickness tears [5].

Various surgical treatments for GTT have been published with a very heterogeneous character [5]. Current techniques, both open and arthroscopic, demonstrate a wide range of retear rates, from 4 % to 37 % [6,7]. This inconsistency in outcomes underscores the need for more effective and reliable treatment approaches.

In response to these challenges, the authors introduce a novel open surgical technique, specifically designed to address Grade III and IV Gluteus Medius (GMed) tendon tears [8]. This innovative approach incorporates augmentation using a polyethylene terephthalate (PET) synthetic ligament, such as the Ligament Advanced Reinforcement System (LARS®). By utilizing a synthetic ligament for augmentation, the authors aim to provide additional support to the repaired tendon during the critical healing phase [[9], [10], [11]].

The medical literature supports the use of open techniques for gluteus medius tendon repair, particularly in cases of a full thickness Grade III or IV tear with severe fatty infiltration where patients may experience greater improvement compared to endoscopic methods. While the specific concept of an artificial ligament in GMed tendon repair is not explicitly detailed in the provided sources, the concept of using artificial ligaments for augmentation in tendon repairs is well-established in orthopedic surgery [11,12].

It is reasonable that an open repair technique for the GMed tendon could be considered for augmentation with an artificial ligament, to enhance the structural integrity and functional outcomes of the repair. Our rationale behind reinforcing a reconstructed GMed Tendon with a synthetic ligament stem, comes from the significantly higher forces it endures, which are over 1000 Newtons at the GMed Tendon compared to the 300–400 Newtons on the Glenohumeral joint. The aim of the current technical report is therefore to provide a detailed description of the surgical procedure for treating these end-staged GTT, mirroring that of repairing a rotator cuff tear in the shoulder.

## 2 Material and methods

The study introduces an innovative open surgical technique for treating Grade III and IV Gluteus Medius (GMed) tendon tears, utilizing a polyethylene terephthalate (PET) synthetic ligament for augmentation.

This prospective, single-center case series comprised 42 consecutive cases, conducted in a private and community setting, to evaluate the surgical technique. This study has been reported in line with the PROCESS criteria [13].

## 3 Surgical technique

The procedure is performed according to following steps:

1. Positioning and Incision

After placing the patient in the lateral position, an incision is made in line with the midline of the femur, dissect down to the iliotibial fascia, and incise the fascia in line with the center of the greater trochanter.

During this stage, if a fluid-filled bursa is present (which is common), fluid emerges when incising the fascia.

2. Exposure and mobilizing the GMed Tendon

**Fig. 2:**
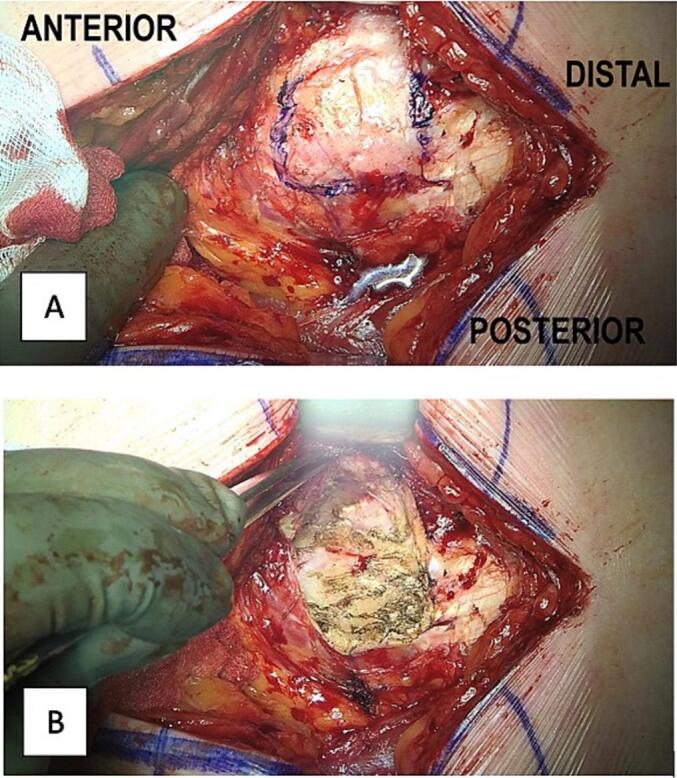
A) Opening the superficial layer (blue border) to enter the deep layers of the GMed. B): Retracted deep layers of the GMed. (For interpretation of the references to color in this figure legend, the reader is referred to the web version of this article.)

Frequently, it is observed that the superficial layer of the gluteus medius is not teared from the insertion site at the Greater Trochanter (GT) [5]. Consequently, an incision is made in the superficial layer to investigate the underlying tissue of the gluteus medius (Fig. 2).

When the gluteus medius is retracted, the tendon should be mobilized from the overlying gluteus maximus and tensor muscles above and the gluteus minimus below by blunt dissection to allow later repair and augmentation.

3. Preparing the insertion of the GT

**Fig. 3:**
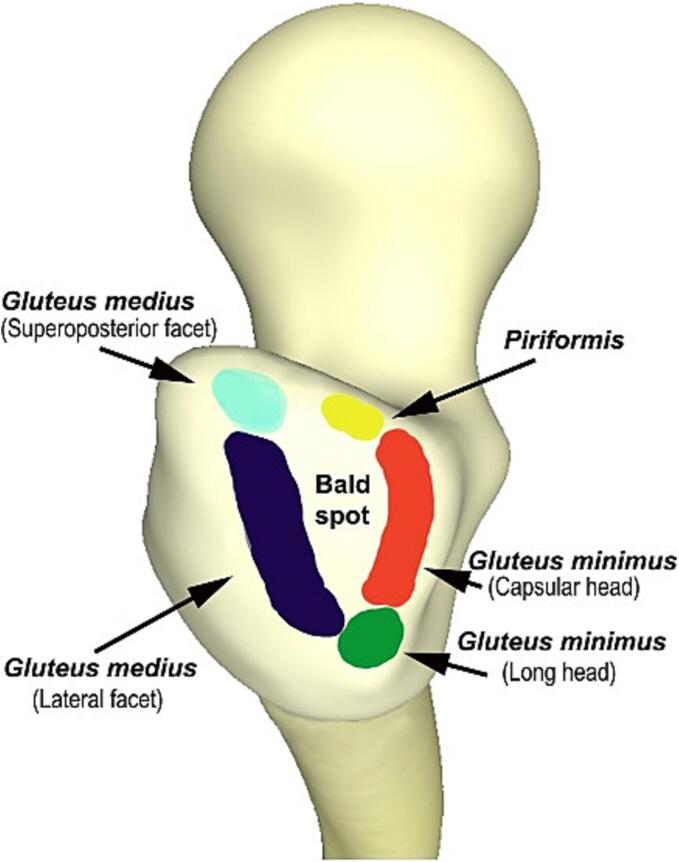
The insertion site of the gluteal medius on the GT.

The GMed tendon has two distinct insertion sites on the greater trochanter: the lateral facet and the superoposterior facet. The posterior part of the gluteus medius tendon converges onto the superoposterior facet, whereas the anterolateral part runs posteroinferiorly toward the lateral facet. This anatomical configuration is crucial for understanding the biomechanics and potential sites of GTPS, such as tears, within the gluteus medius tendon (Fig. 3) [14,15].

The removal of the spurs (Fig. 1) on the anterolateral aspect of the GT is an important technical part of the procedure to prevent later retearing. We use a rongeur or a shizzle to remove these spurs. After this, gently abrade the outer cortical layer of the greater trochanter to stimulate subchondral bleeding, promoting healing. The amount of burring is minimized to prevent excessive removal of the thin cortical bone covering the GT.

4. Transosseous tunnels and knotting

**Fig. 4:**
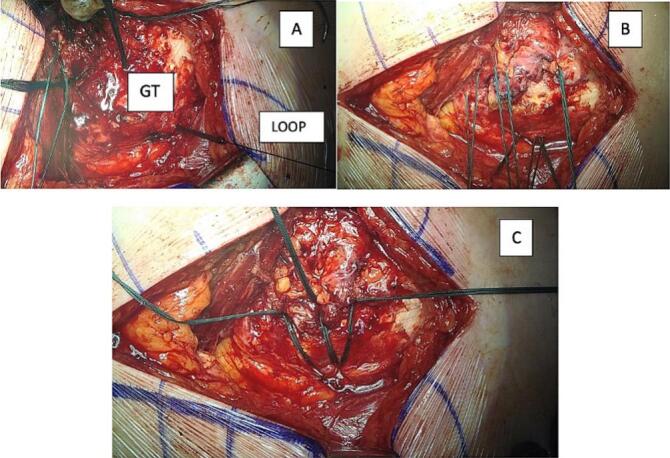
A) Loops are threaded through the GT using a suture retrieving device and a polydioxanone (PDS) loop (indicated) suture B) and C) suturing with non-absorbable.

The Gluteal Tendon is secured using three or four non-absorbable, braided polyester sutures coated with polybutylate (in this case Ethibond™ sutures, size 6) in a double loop configuration. These suture loops should be positioned at the gluteal tendon's insertion point, oriented toward the greater trochanter (GT). Hereto, two or three drill holes (2.8 mm drill is used) are created through the GT and these loops are threaded through the GT. Sometimes 2 loops can go through one hole. This process is facilitated by using a suture retrieving device and a polydioxanone (PDS) loop suture to guide the non-absorbable sutures through the holes (Fig. 4).

The leg is positioned in internal rotation to bring the GMed Tendon to the original insertion site on the GT. We pull the sutures to the insertion using the Nice Knot [16].

The Nice knot, named after the city in France where it was developed, is an innovative suture technique used in orthopedic surgery, particularly for tendon reattachment procedures. This fixation method provides strong and stable anchorage while distributing tension evenly across the repair site. The Nice knot utilizes a sliding-locking mechanism, allowing for precise tensioning and eliminating the need for traditional knot tying [16].

5. Reinforcement with synthetic ligament

After reattaching the gluteal tendon to its insertion point, we enhance and strengthen the reconstruction using a polyethylene terephthalate (PET) synthetic ligament, such as the LARS (Movmedix TM; R06 x 400/6 cm, 4000N strength).

**Fig. 5:**
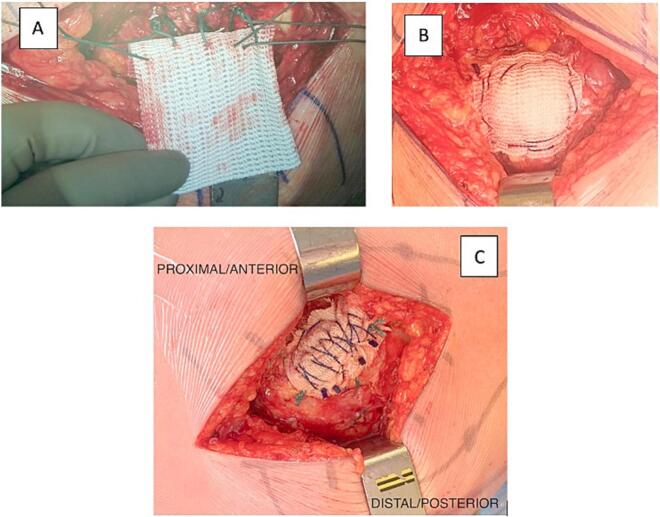
A) Fixation of the synthetic ligament with the ethibond suture B) anchoring the edges of the synthetic ligament and C) transosseous and transtendinous suture with a barbed suture.

This phase of the procedure involves three steps (Fig. 5):

1. The polyethylene terephthalate (PET) synthetic ligament is positioned in the correct position using the previously placed non-absorbable sutures. These sutures go through the synthetic ligament.
2. Following, a type of barbed suture (Stratafix Symmetric PDS Plus, 3.0 from EthiconTM) is employed to anchor the edges of the synthetic ligament, passing through both the GMed tendon and the synthetic ligament.
3. Finally, we attempt to place a transosseous and transtendinous suture in the middle of the synthetic ligament, again using barbed suture.

This multi-step fixation technique provides the reconstruction with the requested stability and strength.

After all these steps we close the fascia over the reconstructed and augmented ligament.

## 4 Postoperative rehabilitation

The postoperative protocol for this procedure is consistent across patients. For 4–6 weeks post-surgery, patients use two crutches while bearing weight on the operated leg. Deep venous thrombosis prevention involves daily Aspirin 160 mg, and patients must avoid active hip abduction. No hip orthosis is used. This approach may differ from other protocols that rely on such devices for additional support or immobilization [10].

The rehabilitation process takes a cautious approach, with physical therapy being initiated no earlier than 6 weeks after the surgery. Once begun, the therapy focuses on two key areas: strengthening the abductor muscles and retraining the patient's gait.

## 5 Preliminary results

Our ongoing study incorporates 42 patients, who were evaluated both operatively and postoperatively using a newly translated and validated Dutch version of the VISA-G scoring system. This assessment tool includes a Visual Analog Scale (VAS) score ranging from 0 to 10 as one of its primary metrics [17].

The study included 36 women and 4 men, average age 65.3 years, with 78 % presenting Grade-III or IV tears. Patient-reported pain decreased from a pre-operative mean VAS score of 7.9 to 2.7 at 6 months, indicating significant and early pain relief. Functional outcomes, measured by VISA-scores, are improving more gradually, suggesting a slower recovery of full function. The one-year follow-up shows no patient loss to date.

## 6 Limitations

Our approach faces several limitations worth acknowledging. A 60-year-old female patient presented with pain, 17 weeks after surgery. Laboratory tests revealed significantly elevated inflammatory markers (CRP 78 mg/L, ESR 33 mm/h) and leukocytosis (11,980/μL with 78 % PMNs), confirming an active infection. The severity of this infection necessitated surgical removal of the affected ligament.

The currently available follow-up period is relatively short, limiting our ability to assess long-term outcomes. Additionally, the small sample demonstrates a significant gender imbalance, with a higher proportion of female participants. This imbalance actually reflects the real-world epidemiology of gluteal tendon tears, which are known to occur more frequently in women.

## 7 Conclusion

Initial outcomes of this novel technique for Gluteal Medius Tendon reinforcement demonstrate significant pain reduction in early postoperative months, with functional recovery progressing more gradually. As a single-center, single-surgeon study, these promising results require validation through multi-center research involving different surgical teams to confirm technique reproducibility and broader clinical applicability.

## Author contribution

This paper is part of a PhD project.

My promotors:

• 2 professors at Ghent University: Guidance in technique development and text composition

Scientific Consulting:

• Professor in Emeritus, Support and guidance on scientific questions

## Informed consent

Written informed consent was obtained from the patients for publication of this case report and any accompanying images. A copy of the written consent is available for review on request.

## Ethical approval

Ethical approval to report this case was obtained from an ethical Board.

## Guarantor

Dr. Van Quickenborne Damien accepts the full responsibility for the work or the conduct of this study.

## Research registration number

1. Name of the registry: Researchregistry.com
2. Unique identifying number or registration ID: Researchregistry10990
3. Hyperlink to your specific registration (must be publicly accessible and will be checked): https://www.researchregistry.com/browse-theregistry#home/registrationdetails/6792068083019802c4e287af/.

## Funding

The authors received no financial support for the research, authorship, and/or publication of this article.

## Conflict of interest statement

The authors declare no potential conflicts of interest with respect to the research, authorship, and/or publication of this article.
